# Estimating the Proportion of True Null Hypotheses for Multiple Comparisons

**Published:** 2008-02-14

**Authors:** Hongmei Jiang, R.W. Doerge

**Affiliations:** 1 Department of Statistics, Northwestern University, 2006 Sheridan Road, Evanston, IL 60208 U.S.A; 2 Department of Statistics, Purdue University, 250 N. University Street, West Lafayette, IN 47907 U.S.A

**Keywords:** false discovery rate, multiple comparisons, type I error rate, microarray, epigenomics

## Abstract

Whole genome microarray investigations (e.g. differential expression, differential methylation, ChIP-Chip) provide opportunities to test millions of features in a genome. Traditional multiple comparison procedures such as familywise error rate (FWER) controlling procedures are too conservative. Although false discovery rate (FDR) procedures have been suggested as having greater power, the control itself is not exact and depends on the proportion of true null hypotheses. Because this proportion is unknown, it has to be accurately (small bias, small variance) estimated, preferably using a simple calculation that can be made accessible to the general scientific community. We propose an easy-to-implement method and make the R code available, for estimating the proportion of true null hypotheses. This estimate has relatively small bias and small variance as demonstrated by (simulated and real data) comparing it with four existing procedures. Although presented here in the context of microarrays, this estimate is applicable for many multiple comparison situations.

## Introduction

Genomic technologies are producing vast amounts of biological data that are the basis for investigations that require repetitive testing of the same hypothesis. Because the number of tests performed (e.g. differential expression) is so large, sometimes the multiple comparison procedures that control the familywise error rate are too strict for biological applications (e.g. differential methylation). In fact, many biologists would rather experience several more false positives (i.e. type I errors; false rejections of the null hypothesis) than lose important information. In an attempt to address the multiple comparison issue Benjamini and Hochberg (1995) introduced an error rate measure called False Discovery Rate (FDR). Specifically, a family of *m* hypothesis tests is considered, of which *m*_0_ are true. The proportion of erroneously rejected null hypotheses among all the rejected null hypotheses can be cap tured by the random variable *Q* = *V/R*,where *R* is the number of rejected hypotheses and *V* is the number of false rejections (type I errors). Benjamini and Hochberg (1995) formally define the FDR to be the expected proportion of falsely rejected hypotheses among all the rejections,

(1) FDR=E(Q)=E(V/R),

where *Q* = 0 when *R* = 0 (no rejections).

If we let *p*_(1)_ ≤ *p*_(2)_ ≤ *···* ≤ *p*_(_*_m_*_)_ be the ordered p-values and *H*_(_*_i_*_)_ be the null hypothesis corresponding to *p*_(_*_i_*_)_, then in Benjamini and Hochberg’s (BH) FDR controlling procedure (Benjamini and Hochberg, 1995), K is considered to be the largest k such that *p*_(_*_k_*_)_ ≤ (*k/m*)*α*, where *α* is the pre-chosen FDR significance level. If K exists, all null hypotheses *H*_(_*_i_* _)_*, i* = 1*, ··· ,K* are rejected. If no such K exists, then no hypotheses are rejected. The BH FDR controlling procedure controls the FDR at exactly the level (*m*_0_*/m*)*α* ≤ *α*, and hence conservatively at *α* , for independent test statistics and for any configuration of false null hypotheses (Benjamini and Yekutieli, 2001; Storey, et al. 2004). In 2000 Benjamini and Hochberg proposed an adaptive procedure which provides more power than the original FDR controlling procedure by comparing each *p*_(_*_k_*_)_ with (*k/m̂*_0_)*α* where *m̂*_0_ is an estimate of *m*_0_. If the estimated value of *m*_0_ is such that *m̂*_0_ ≥ *m*_0_ with probability one, then the adaptive BH FDR controlling procedure will lead to $FDR=\frac{m_{0}}{m}\left(\frac{m}{{\hat{m}}_{0}}\alpha \right)=\frac{m_{0}}{{\hat{m}}_{0}}\alpha \leq \alpha$. Because the accuracy and variation of the estimate of *m*_0_, or *π*_0_ = *m*_0_*/m*, directly affects the performance of the adaptive FDR controlling procedure our focus is on the estimation and effect of *π*_0_.

We propose a simple and easy-to-implement method for estimating the proportion of true null hypotheses. The performance of this estimate is compared to existing methods via simulated and real data. Specifically, Benjamini and Hochberg (2000) estimated the number of true hypotheses from the observed p-values using the Lowest SLope (LSL) estimator. Their approach was based on a modification of the graphical method of Schweder and Spjotvoll (1982). Alternatively, Storey (2002) proposed an estimate of π_0_ by assuming the p-values corresponding to true null hypotheses are uniformly distributed on the interval (0,1) and selecting a reasonable tuning parameter 0 ≤ *λ* < 1. Finally, Langaas, et al. (2005) derived estimators based on nonparametric maximum likelihood estimation of the p-value density, under the restriction of decreasing and convex decreasing densities. Although Benjamini and Hochberg’s original and adaptive FDR controlling procedure are developed for independent statistics these procedures can also be applied to some dependence structures (Benjamini and Yekutieli, 2001). Simulations have also demonstrated that they can be used for situations where there is a weak correlation structure among the genes (Storey et al. 2004). However, because of the small number of biological replicates used in most micro-array experiments, it is very difficult to measure the correlation structure among a set or family of genes. Reiner et al. (2003) proposed a procedure for the general case, but it is conservative when compared to the adaptive FDR controlling procedures.

## Methods

### Storey’s approach

Our approach is motivated by the work of Storey (2002), where the proportion of true null hypotheses, *π*_0_, is estimated by

(2) $${\hat{\pi }}_{0}(\lambda )=\frac{W(\lambda )}{(1-\lambda )m},$$

where *W*(*λ*) = #{*p_i_* : *p_i_* > *λ*}, *and* 0 ≤ λ < 1 is a tuning parameter. The rationale for this estimate is that p-values corresponding to true null hypotheses are uniformly distributed on the interval (0,1), of which most should be close to 1. Thus, for a reasonable λ, there are about *m*_0_(1 – λ) such p-values in the interval (*λ,*1] such that *W*(*λ*) ≈ *m*_0_(1 – *λ*). Black (2004) pointed out that Equation (2) is an unbiased estimate of π_0_ for all values of *λ* if all the null hypotheses are true and the p-values have a uniform distribution on the interval (0,1). However, there is an upward bias when the p-values come from both true null and true alternative hypotheses. As it turns out, choosing the tuning parameter *λ* in Equation (2) is very important since there is a bias-variance trade-off. When *λ →* 0, the variance of *π̂*_0_ (*λ*) becomes smaller and the bias of this estimate increases. When *λ →* 1, the bias of *π̂*_0_ (*λ*) becomes smaller, and the variance of this estimate increases. To address this point, Storey et al. (2004) proposed a bootstrap method that automatically chooses *λ* when estimating *π̂*_0_ (*λ*).

Instead of choosing one specific *λ*, Storey and Tibshirani (2003) proposed an estimate of *π*_0_ using $\underset{\lambda \to 1}{\text{lim}}{\hat{\pi }}_{0}(\lambda )$ so that the bias is small and there is a balance between both bias and variance. For this approach, *π̂*_0_ (*λ*) is plotted over a range of *λ* = 0,0.05,0.10,*…,*0.90 and then a natural cubic smoothing spline is fit to these data for the purpose of estimating the overall trend of *π̂*_0_ (*λ*) as *λ →* 1. In the QVALUE (http://faculty.washington.edu/~jstorey/) software, the predicted value of *π̂*_0_ (*λ*) at *λ* = 0.90 is chosen as the estimate of *π*_0_.

### Average estimate approach

As mentioned previously, the estimate ${\hat{\pi }}_{0}(\lambda )=\frac{W(\lambda )}{(1-\lambda )m}$ where 0 ≤ *λ* <1, has a large bias and small variance when *λ* is small and a small bias and large variance when *λ* is big. Suppose for each *λ_i_*,where 0 < *λ*_1_ < *λ*_2_ < *···* < *λ_n_* < 1, we compute *π̂*_0_ (*λ_i_*) as in Equation (2), then

$$E[{\hat{\pi }}_{0}(\lambda_{i})]=\pi_{0}+{ɛ}_{i},$$

where *E*[*ɛ_i_*] = *δ_i_*, *δ_i_* *≥ δ_i_*_+1_, *Var* [*ɛ_i_*] = *σ_i_*^2^, and *σ_i_*^2^*≤σ_i_*_+_*_1_*^2^. Therefore, a natural choice is to consider the average of *π̂*_0_(*λ*) over the values of *λ_i_*

$${\hat{\pi }}_{0}=\frac{1}{n}\sum_{i=1}^{i=n}{\hat{\pi }}_{0}(\lambda_{i}).$$

The bias of *π̂*_0,_ 1/*_n_*∑*_i_*_=1_*^i^*^=n^ , is smaller than *δ*_1_ (the bias of the estimate of *π*_0_ at *λ* = *λ*_1_) and at the same time, *π̂*_0_ has a smaller variance. Considering the average of *π̂*_0_ (*λ*) over a range of *λ* to estimate *π*_0_ reduces the problem to choosing the range of *λ*.

Define 0 = *t*_1_ < *t*_2_ <*···* < *t_B_* < *t_B_*_+1_ = 1 as equally spaced points in the interval [0,1] such that the interval [0,1] is divided into *B* small intervals with equal length 1*/B*. Specifically, *t_i_* = (*i–*1)*/B.* For example, when *B* = 10, *t*_1_ = 0*, t*_2_ = 0.1,*…, t*_10_ = 0.9. For each *t_i_* (*i=* 1,*…,B*), *π̂*_0_ (*t_i_*) is an estimate of π_0_ via Equation (2) with *λ* = *t_i_*. The goal then becomes finding a subset of *t_i_*’s such that a new estimate of π_0_ is obtained by taking the average of the corresponding values of *π̂*_0_ (*t_i_*). Let *NB_i_* denote the number of p-values which are greater than or equal to *t_i_*, and let *NS_i_* represent the number of p-values in the interval of [*t_i_,t_i_*_+ 1_). Therefore,

(3) $${NB}_{i}=\#\{p_{k}:p_{k}\geq t_{i}\},$$

(4) $${\hat{\pi }}_{0}(t_{i})=\frac{{NB}_{i}}{(1-t_{i})m}$$

(5) $${NS}_{i}=\#\{p_{k}:t_{i}\leq p_{k}<t_{i+1}\},$$

where *i* = 1,*…,B*.

If the *NB_i_* p-values come from the null distribution, then on average there are $\frac{{NB}_{i}}{B-i+1}$ p-values in each of the (*B–i* + 1) small intervals on [*t_i_,*1]. In other words, there are $\frac{{NB}_{i}}{B-i+1}$ p-values in each small interval [*t_j_,t_j_*_+1_) for *i* ≤ *j* ≤ *B*. Since the p-values corresponding to the true alternative hypotheses are smaller than those corresponding to the true null hypotheses, there are more p-values in the intervals [*t_i_* *,t_i+_*_1_) with small index *i*. For small *i*, *NS_i_* is usually greater than $\frac{{NB}_{i}}{B-i+1}$. Therefore, initiating from *i* = 1, we find the first *i* such that ${NS}_{i}\leq \frac{{NB}_{i}}{B-i+1}$. If such *i* exists, *t_i_* can be considered as the change point and we assume all the p-values bigger than *t_i_* come from the true null hypotheses. From this *π*_0_ can be estimated by

(6) $${\hat{\pi }}_{0}(B)=\frac{1}{B-i+1}\sum_{j=i}^{j=B}{\hat{\pi }}_{0}(t_{j})$$

(7) $$=\frac{1}{B-i+1}\sum_{j=i}^{j=B}\frac{NB_{j}}{(1-t_{j})m}$$

where $i=\text{min}\{i:NS_{i}\leq \frac{{NB}_{i}}{B-i+1}\}$. In order to find the range of *λ*, only a lower bound of *λ* is required. The large values of *t_i_* are used so that it ensures the estimate of *π*_0_ has small bias. This is equivalent to fitting a straight line with slope 0 in the right bottom part of a *π̂*_0_ (*t_i_*) versus *t_i_* plot, such that the intercept provides the estimate of *π*_0_. A simple modification of this approach is to estimate *π*_0_ by taking the average of *π̂*_0_(*t_j_*) from *j* = *i–*1 to *B*, that is,

(8) $${\hat{\pi }}_{0}(B)=\frac{1}{B-i+2}\sum_{j=i-1}^{j=B}\frac{NB_{j}}{(1-t_{j})m}$$

where $i=\text{min}\{i:NS_{i}\leq \frac{{NB}_{i}}{B-i+1}\}$. This ensures that the upward bias increases and the variance decreases, as *π̂*_0_ (*t_i_* _–1_) has smaller variance and bigger bias than *π̂*_0_ (*t_j_*) for *j* = *i,…, B*.

A remaining challenge is how to choose B. Specifically, how many *λ*’s should be used in the interval [0,1]. Recall that a motivating factor of the proposed average estimate approach is to balance the bias and variance. The natural way to measure both the bias and variance is the mean-squared error, *E*[*π̂*_0_ (*B*)−*π*_0_]_2_. Since the true value of *π*_0_ is unknown and the theoretical result is intractable, we take a bootstrap approach in the following way:

1. For each *B* ε *I*, *I* = {5, 10, 20, 50, 100}, compute [*π̂*_0_ (*B*) as in Equation (8).
2. Form *N* bootstrap samples of the p-values, and compute the bootstrap estimates [*π̂*_0_^*^^b^(*B*) for *b* = 1,*…, N* and *B* ε {5, 10, 20, 50, 100}.
3. For each *B* ε *I*, estimate its respective mean-squared error as

$$\hat{MSE}(B)=\frac{1}{N}\sum_{b=1}^{N}{\left[{\hat{\pi }}_{0}^{*b}(B)-{\bar{\pi }}_{0}\right]}^{2},$$

Where

$${\bar{\pi }}_{0}={\text{average}}_{B^{\prime }\in I}\{{\hat{\pi }}_{0}(B^{\prime })\}$$

1. Let $\hat{B}=\text{arg}{\text{min}}_{B\in I}\hat{MSE}(B)$, then the estimate of *π*_0_ *is π̂*_0_ = *π̂*_0_ (*B̂*).

Notice that in step three the value of *π*_0_ is estimated by the average of the *π̂*_0_ (*B*) over arrange of *B*.

## Results

### Simulation studies

To investigate the performance of the proposed average estimate approach, a simulation study was performed. Taking *m* = 1,000 (i.e. 1,000 genes are tested for differential expression), let *π*_0_ vary over a wide range, say *π*_0_ = 0.50,0.60,*…,*0.90 which are reasonable for microarray experiments. Hypotheses, *H*_0_: μ = 0 versus *H_a_*: μ > 0, are tested for independent random variables *Z_i_* (*i* = 1,*…,m*) from null distribution N(0,1) and alternative distribution N(2,1). Specifically, *mπ*_0_ and *m*(1 *–π*_0_) random variables have mean 0 and 2, respectively. For each test, the p-value is computed as *p_i_* = *P*(*Z* > *z_i_*), where *Z* is a random variable from a standard normal distribution N(0,1) and *z_i_* is the observed value of *Z_i_*. For each value of *π*_0_, *l* = 1,000 data sets were simulated.

For the choice of B, *B* is either fixed (i.e. *B* = 5, 10, 20, 50, and 100) or chosen by the proposed bootstrap approach. For each of the *l* = 1,000 simulated data sets, when *B* is fixed, the estimate of *π*_0_ is computed via Equation (8), that is, ${\hat{\pi }}_{0}=\frac{1}{B-i+2}\Sigma_{j=i-1}^{j=B}{\hat{\pi }}_{0}(t_{j})$ where $i=\text{min}\{i:{NS}_{i}\leq \frac{{NB}_{i}}{B-i+1}\}$. If such *i* does not exist, *π*_0_ is estimated by the average of *π̂*_0_ (*t_B_* _–1_)and *π̂*_0_ (*t_B_*). For the bootstrap approach to automatically choose *B*, the range of *B* is 5, 10, 20, 50, 100.

For completion the performance of the proposed average estimate approach is compared with several existing procedures:

1. Benjamini and Hochberg’s lowest slope estimate (LSL) (Benjamini and Hochberg, 2000),
2. Storey’s bootstrap estimate (Storey_boot_) (Storey et al. 2004),
3. Storey and Tibshirani’s smoother estimate (ST_smoother_) (Storey and Tibshirani, 2003),
4. Langass et al.’s nonparametric maximum likelihood estimate (convest) (Langaas et al. 2005).

For procedures 2 and 3, the QVALUE software (http://faculty.washington.edu/~jstorey/) was employed. For procedure 4, the R function ‘convest’ was downloaded from the R library ‘limma’ as part of the Bioconductor project (http://www.bioconductor.org).

Table 1 summarizes the simulation results. Bias and the standard deviation of the estimates are estimated by

$$\begin{array}{l} \hat{Bias}=\frac{1}{l}\sum_{i=1}^{i=l}\left({\hat{\pi }}_{0i}-\pi_{0}\right)\\ \hat{Std}=\sqrt{\frac{1}{l-1}{\sum_{i=1}^{i=l}\left({\hat{\pi }}_{0i}-\frac{1}{l}\sum_{i=1}^{i=l}{\hat{\pi }}_{0i}\right)}^{2}}, \end{array}$$

where *π̂*_0_*_i_* estimates *π*_0_ for the *i* th simulation, and *π*_0_ is the true value. As demonstrated, the LSL approach has the largest upward bias which guarantees that Benjamini and Hochberg’s adaptive FDR controlling procedure controls the FDR below a pre-chosen FDR level. However, the FDR can be much lower than the pre-chosen FDR level. The LSL approach also has the smallest variation. The last three approaches [2–4] all underestimate the proportion of true null hypotheses. The proposed average estimate approach provides estimates of π_0_ that have upward but relatively small bias and relatively small variance regardless of whether *B* is fixed or automatically chosen via bootstrap procedure. When *B* increases, the bias increases and the variation decreases. Both the small upward bias and small variance provide evidence that the proposed average estimate approach has better properties when compared to the other approaches.

**Figure 1:**
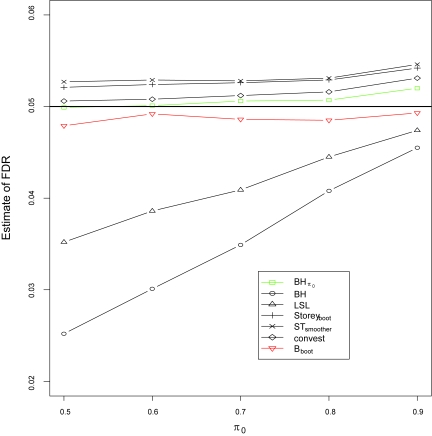
Simulation results of the False Discovery Rate (FDR) at significance level *α* = 0.05 for seven procedures: Benjamini and Hochberg’s FDR controlling procedure with incorporation of the true π_0_ (BHπ_0_ ), Benjamini and Hochberg’s FDR controlling procedure (BH), Benjamini and Hochberg’s adaptive approach with incorporation of the estimate of π_0_ which is estimated by the proposed average estimate procedure where *B* is chosen via bootstrapping (B_boot_), Benjamini and Hochberg’s lowest slope approach (LSL), Storey’s bootstrapping approach (Storey_boot_), Storey and Tibshirani’s smoother method (ST_smoother_), and Langass et al.’s nonparametric maximum likelihood estimate (convest), respectively. The black straight line represents FDR = 0.05. The total number of hypotheses tests is *m* = 1, 000 and the size of simulation study 1,000 for each value of π_0_.

The average of the true false discovery rate (FDR) from 1000 simulations is also compared in this simulation study by applying Benjamini and Hochberg’s adaptive FDR controlling procedure (Benjamini and Hochberg, 2000) with *π*_0_ estimated using the above mentioned five methods (Fig. 1). The FDR significance level was chosen as *α* = 0.05. For the purpose of comparison, the original BH FDR controlling procedure (Benjamini and Hochberg, 1995) and the adaptive FDR controlling procedure with the incorporation of the true value of *π*_0_ were also applied to the p-values. It can be seen that the original BH FDR controlling procedure has the lowest FDR as expected. Because Benjamini and Hochberg’s lowest slope approach overestimates *π*_0_, the FDR is below, but much lower than, the pre-chosen *α*, although this approach has a bigger FDR than that of the BH procedure. Storey’s bootstrap estimate, the smoother estimate and convest estimate produce higher FDRs than the pre-chosen level because all three methods underestimate *π*_0_. Our proposed average estimate approach overestimates *π*_0_, its FDR is below but very close to the pre-chosen significance level *α* = 0.05. Table 1 also demonstrates that the FDR for the proposed average estimate has the relatively small variation.

**Figure 2:**
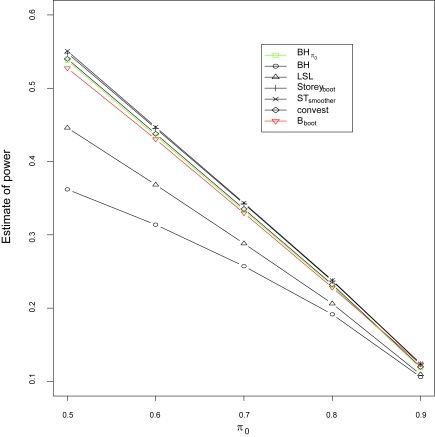
Simulation results for the evaluation of statistical power at significance level *α* = 0.05 for seven procedures: Benjamini and Hochberg’s FDR controlling procedure with incorporation of the true π_0_ (BHπ_0_ ), Benjamini and Hochberg’s FDR controlling procedure (BH), Benjamini and Hochberg’s adaptive approach with incorporation of the estimate of π_0_ which is estimated by the proposed average estimate procedure where *B* is chosen via bootstrapping (B_boot_), Benjamini and Hochberg’s lowest slope approach (LSL), Storey’s bootstrapping approach (Storey_boot_), Storey and Tibshirani’s smoother method (ST_smoother_) and Langass et al.’s nonparametric maximum likelihood estimate (convest), respectively. The total number of hypotheses tests is *m* = 1, 000, and the size of simulation study is 1,000 for each value of π_0_.

The power of the five adaptive FDR controlling procedures is compared (Fig. 2). The power of a procedure is measured by average power which is defined to be the ratio of average number of correct rejections of true alternative hypotheses to the total number of true alternative hypotheses. Formally, *average power* = *E*(*S*)*/*(*m – m*_0_). As illustrated, the power decreases when *π*_0_ increases for all of the FDR controlling procedures. The original BH procedure has the lowest power, while Benjamini and Hochberg’s adaptive procedure has the second lowest power. It is not surprising that Storey_boot_ procedure has the largest statistical power, because the FDR of this procedure exceeds the pre-chosen FDR significance level (Fig. 1).

### Microarray data application

The same five estimating π_0_ methods were also applied to the training samples of the leukemia data of Golub et al. (1999), which consist of 27 patients with acute lymphoblastic leukemia (ALL) and 11 patients with acute myeloid leukemia (AML). The samples were assayed using Affymetrix Hgu6800 chips and the gene expression data of 7129 genes (Affymetrix probes) are available from R library golubEsets. For each gene, a simple two-sample t-test was employed for testing differential gene expression and the p-value was computed. Table 2 gives the estimate of the proportion of true null hypotheses and the number of statistically significant genes.

From this real data analysis, it can be seen that the Benjamni and Hochberg’s LSL approach conservatively overestimates *π*_0_, hence it leads to lowest power in terms of the number of rejections. Our proposed average approach provides a slightly larger estimate than Storey’s bootstrap approach, the smoother estimate and the nonparametric maximum likelihood approach (convest), even though they end up with a similar number of rejections.

## Summary

As array technology improves, it is anticipated that the number of features per array will only increase, hence multiple comparisons will continue to be a challenging problem. Specific to microarrays, the false discovery rate (FDR) is preferred to family-wise error rate (FWER) because the FDR controlling procedures have more statistical power than the FWER controlling procedures, even at the cost of a few more type I errors (i.e. false positives). Since Benjamini and Hochberg (1995) proposed their FDR controlling procedure, a variety of methods have been proposed to estimate *π*_0_, the proportion of true null hypotheses. As seen here, overestimating π_0_ controls the FDR below the specified rate. When our and others, estimate of π_0_ is incorporated into the Benjamini and Hochberg’s FDR controlling procedure, the adaptive FDR controlling procedure has more power and an FDR close to the pre-chosen level.

In this work, we have compared several methods for estimating the proportion of true null hypotheses (*π*_0_). Benjamini Hochberg’s lowest slope approach (Benjamini and Hochberg, 2000) overestimates *π*_0_. Storey’s estimate *π̂*_0_(*λ*) (Storey, 2002) also overestimates *π*_0_ for any fixed value 0 ≤ *λ* <1. When *λ →* 1, the bias becomes smaller, and the variance becomes bigger. In order to find the optimal *λ* such that *π̂*_0_(*λ*) has small variation, Storey proposed a bootstrapping method (Storey et al. 2004). However, this method underestimates *π*_0_ and the downward bias increases as the true value *π*_0_ gets bigger. Storey and Tibshirani (2003) proposed a smoother method to estimate $\underset{\lambda \to 1}{\text{lim}}{\hat{\pi }}_{0}(\lambda )$ such that this estimate has small bias. Unfortunately, this method also underestimates *π*_0_, although the bias is very small. Furthermore, the variation of this estimate is relatively large, which makes the adaptive FDR controlling procedure unstable. More recently, Langaas et al. (2005) proposed an estimate based on the nonparametric maximum likelihood function of the p-value density restricted to convex decreasing densities. However, this method also underestimates *π*_0_, most likely because the distribution of the p-values is not decreasing for large p-values and tends to be flat.

Using the limitations of the existing approaches for estimating *π*_0_ as the motivation, we propose the average estimate approach by taking average of the estimates of *π*_0_ over a range of equally spaced points on the interval [0,1]. While our average estimate approach has a slightly larger bias, it also has smaller variation than any of the other methods. Furthermore, when compared to the other methods it is easy to implement (e.g. Excel) when the number of points used in approach is fixed (say, *B* = 10), and can be automated to choose *B* via a bootstrap procedure (R code available: www.stat.purdue.edu/~doerge). When our proposed estimated value of *π*_0_ is incorporated into Benjamini and Hochberg’s adaptive FDR controlling procedure, more statistical power is gained such that the FDR can be controlled below, yet extremely close to a desired level *α*.

## Figures and Tables

**Table 1:** The estimate of the proportion of true null hypotheses is compared for: Benjamini and Hochberg’s lowest slope approach (LSL), Storey’s *π̂*_0_ (*λ*) estimate with *λ* selected via bootstrapping (Storey_boot_), Storey and Tibshirani’s smoother method (ST_smoother_), Langass’s nonparametric maximum likelihood approach (convest), and the proposed average estimate approach with fixed values of *B* = 5, 10, 20, 50, 100 and with *B* chosen via the bootstrapping procedure (*B*_boot_). There are 1,000 simulated data sets, each with a total of *m* = 1, 000 hypothesis tests, for each value of *π*_0_.

π_0_	0.5	0.6	0.7	0.8	0.9
	Estimates of π_0_				
LSL	0.7151	0.7889	0.8561	0.9184	0.9683
Storey_boot_	0.4814	0.5789	0.6765	0.7728	0.8660
ST _smoother_	0.4951	0.5939	0.6980	0.7993	0.8973
convest	0.4963	0.5938	0.6947	0.7921	0.8882
*B* = 5	0.5132	0.6113	0.7136	0.8086	0.9058
*B* = 10	0.5082	0.6084	0.7083	0.8045	0.9052
*B* = 20	0.5141	0.6128	0.7115	0.8076	0.9064
*B* = 50	0.5196	0.6175	0.7156	0.8106	0.9078
*B* = 100	0.5243	0.6210	0.7180	0.8122	0.9085
*B***boot**	0.5195	0.6175	0.7148	0.8113	0.9082

	**Standard deviation of π_0_ estimates**				
LSL	0.0323	0.0269	0.0225	0.0155	0.0092
Storey_boot_	0.0467	0.0491	0.0513	0.0522	0.0549
ST _smoother_	0.0513	0.0570	0.0608	0.0654	0.0656
convest	0.0331	0.0364	0.0337	0.0321	0.0328
*B* = 5	0.0335	0.0356	0.0420	0.0428	0.0382
*B* = 10	0.0391	0.0390	0.0402	0.0412	0.0366
*B* = 20	0.0331	0.0343	0.0358	0.0371	0.0331
*B* = 50	0.0293	0.0309	0.0321	0.0334	0.0315
*B* = 100	0.0272	0.0291	0.0307	0.0321	0.0312
*B*_boot_	0.0301	0.0301	0.0313	0.0313	0.0311

**Table 2:** The estimate of the proportion of true null hypotheses and the number of statistically significant genes for the leukemai data (Golub et al. 1999) at significance level *α* = 0.05 after applying Benjamni and Hochberg’s adaptive FDR controlling procedure with *π*_0_ estimated using five methods: Benjamini and Hochberg’s lowest slope approach (LSL), Storey’s *π̂*_0_ (*λ*) estimate with *λ* selected via bootstrapping (Storey**boot**), Storey and Tibshirani’s smoother method (ST**smoother**), Langass’s convest approach (convest), and the proposed average approach with *B* chosen via the bootstrapping procedure (*B***boot**). A two-sample t-test was used to compute the p-values.

Method	Estimate of π_0_	Number of Signicant genes
LSL	0.899	584
Storey_boot_	0.595	787
ST _smoother_	0.583	791
convest	0.595	787
*B*_boot_	0.604	776
